# DNPcall: a new pipeline for accurate double nucleotide polymorphism calling

**DOI:** 10.1093/bioadv/vbaf209

**Published:** 2025-09-05

**Authors:** Letizia Pistacchia, Francesco Ravasini, Elisa Bella, Eugenia D’Atanasio, Fulvio Cruciani, Beniamino Trombetta

**Affiliations:** Department of Biology and Biotechnologies “C. Darwin”, Sapienza University of Rome, Rome, 00185, Italy; Institute of Molecular Biology and Pathology, CNR, Rome, 00185, Italy; Department of Biology and Biotechnologies “C. Darwin”, Sapienza University of Rome, Rome, 00185, Italy; Centre for Palaeogenetics, Stockholm, SE-106 91, Sweden; Department of Biology and Biotechnologies “C. Darwin”, Sapienza University of Rome, Rome, 00185, Italy; Institute of Molecular Biology and Pathology, CNR, Rome, 00185, Italy; Institute of Molecular Biology and Pathology, CNR, Rome, 00185, Italy; Department of Biology and Biotechnologies “C. Darwin”, Sapienza University of Rome, Rome, 00185, Italy; Institute of Molecular Biology and Pathology, CNR, Rome, 00185, Italy; Department of Biology and Biotechnologies “C. Darwin”, Sapienza University of Rome, Rome, 00185, Italy

## Abstract

**Motivation:**

Among the genomic variants of the human genome, Double Nucleotide Polymorphisms (DNPs) are still understudied. They consist of two adjacent variant nucleotides that arise from a single mutational event. Despite their potential relevance in the study of genetic variation, no method currently exists to directly and reliably call DNP genotypes at the individual level.

**Results:**

We present DNPcall, a new pipeline for accurately genotyping putative DNPs based on the pileup file obtained with samtools. DNPcall leverages the information about the read name to finely reconstruct the genotype of the DNPs at the individual level. The genotype is called when both positions of the DNPs are covered by the same read, ensuring that no spurious calls due to sequencing errors are included. In this way, DNPcall can also discriminate between DNPs arising by a single mutation and two adjacent SNPs. The latter ones will indeed result in spurious calls of the putative DNP because the two alternative variants are not linked.

**Availability and implementation:**

DNPcall is a user-friendly pipeline designed to enhance the study of genomic variation. It can also be adapted and implemented to study other kinds of Multi Nucleotide Variants (MNVs) or, in general, microhaplotypes. Source code and documentation are available at https://github.com/fravasini/DNPcall.

## 1 Introduction

Genetic variants are fundamental to understand population dynamics and the mechanisms underlying genomic diversity. Although Single Nucleotide Polymorphisms (SNPs) are the most common, and currently the most studied variants for genetic diversity inferences, Multi Nucleotide Variants (MNVs) may provide additional layers of complexity and valuable insights (Wang *et al.* 2020). Among MNVs, the most simple case is represented by Double Nucleotide Polymorphisms (DNPs), which are defined as two adjacent variant positions occurring on the same haplotype in one individual and are characterized by unique biological and population genetic properties (Rosenfeld *et al.* 2010).

Two adjacent variant positions can emerge through two distinct mechanisms: (i) independent mutational events in the two bases occurring at separate times and therefore in different individuals; or (ii) a single mutational step caused by a replication error of the error-prone polymerase zeta (Rosenfeld *et al.* 2010, Wang *et al.* 2020). These mechanisms give rise to two types of molecular variants, with very distinct properties. The first type, formed through independent mutations, is characterized by the potential presence of three haplotypes within the population (barring recombination and recurrent mutations), because the two SNPs do not share a common origin. In this case, the derived alleles of the single bases are rarely found in the same haplotype, being actually two adjacent SNPs. In contrast, the second mechanism will generate real DNPs, distinct from the other types of variants at the population level. Excluding very rare recombination events that might occur between the two bases, only two haplotypes can be found within the population: the ancestral and the derived one. As a consequence, the two variant positions in the DNP always exhibit identical allele frequencies across the population. On the contrary, two adjacent SNPs that arise through independent mutations (false DNPs), usually show distinct allele frequencies (Wang *et al.* 2020). For their characteristics, DNPs can be easily inferred at the population level. The two adjacent variant positions are usually called independently and only subsequently the microhaplotype they are representing is reconstructed, based on allele frequencies. To our knowledge, at the moment there is no pipeline or software to specifically call DNPs at the individual level, considering them as unique markers rather than “adjacent SNPs,” and therefore to actually reconstruct the DNP state instead of just inferring it. In this paper we present a pipeline to call together and in a reliable way the two variant positions of a DNP: DNPcall (https://github.com/fravasini/DNPcall).

## 2 Methods

### 2.1 Implementation

**Figure 1.**
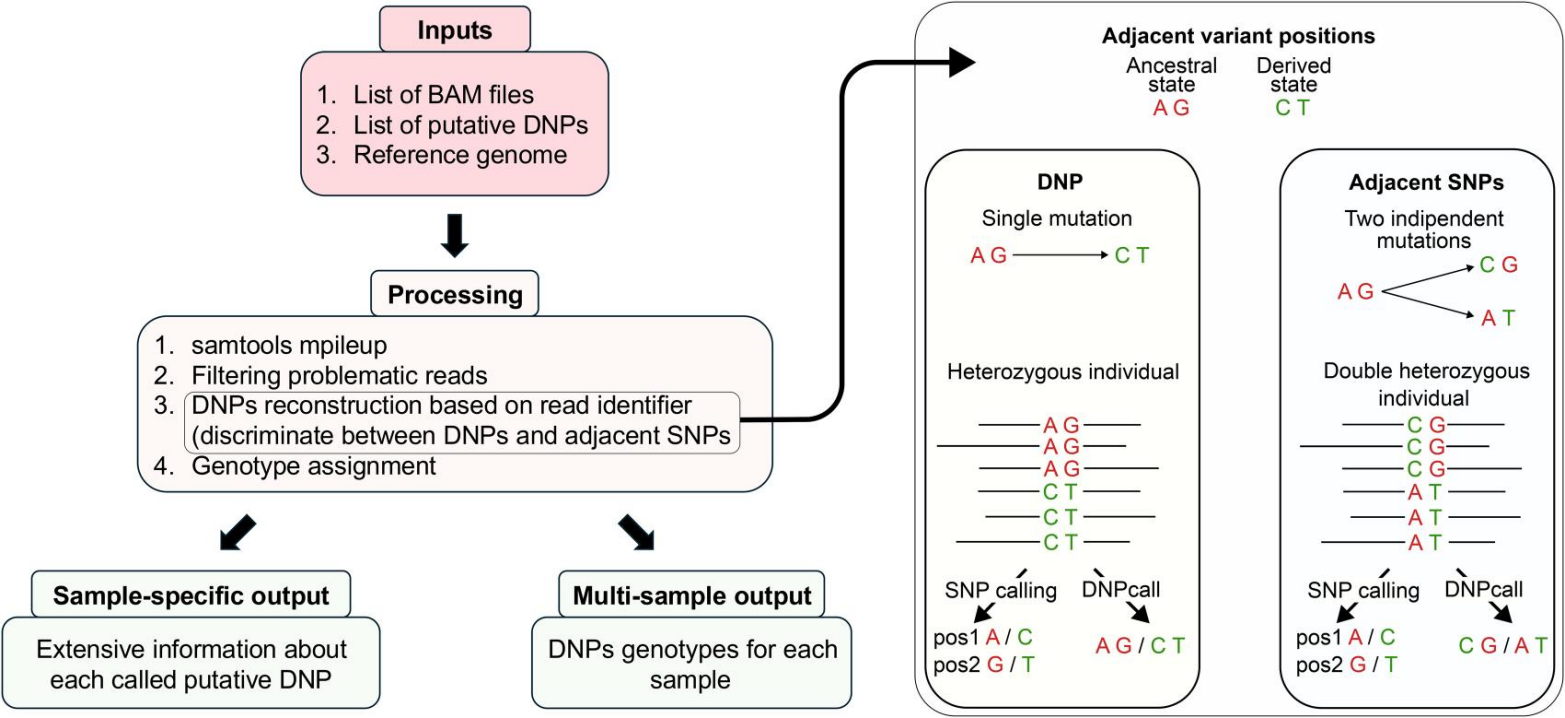
On the left, schematic representation of DNPcall workflow. The process of DNP reconstruction is illustrated on the right with an example. Two adjacent nucleotide positions may change from an ancestral to a derived state either due to a single mutation (DNP) or two independent mutations occurring in different individuals (adjacent SNPs). In a heterozygous individual, the distribution of derived and ancestral bases in sequencing reads differs: for a DNP, both derived bases appear together in the same reads, whereas for adjacent SNPs, the derived alleles are present in separate reads. Standard SNP calling cannot distinguish between these two scenarios, whereas DNPcall performs a precise reconstruction, allowing to discriminate between DNPs and adjacent SNPs. The extremely rare case of two independent mutations occurring in two adjacent positions on the same chromosome cannot be disentangled with a bioinformatic approach. These cases can only be discovered with a thorough analysis of the allele frequencies in the two independent positions at the population level.

DNPcall performs DNP calling on individual alignment files in BAM format considering the two variant positions together, instead of calling the single positions and implicitly assuming that they belong to the same haplotype. This step is essential for accurate DNP identification, as recombination events or sequencing errors could result in false positives, mistakenly identifying DNPs that are actually single variants located on different homologous chromosomes. DNPcall exploits the read names of the single variant positions to finely reconstruct the DNP represented, because a read is always deriving from a single chromosome. To do so, DNPcall leverages the option --output-QNAME of samtools mpileup command which returns the read identifier for each position. Later, the DNP is reconstructed by grouping together the positions with the same read identifier. In this way, the variants obtained are undoubtedly from the same chromosome and the genotypes are called correctly (Fig. 1).

Other measures implemented in DNPcall are also necessary to perform a correct DNP calling. Sequencing errors may introduce indels in the reads and these will affect the DNP calling, possibly leading to an allelic imbalance. Therefore, all the reads containing indels in the DNP positions are discarded to avoid this problem. Moreover, indels induced by sequencing errors outside the two positions of the DNP may cause incorrect genotypes assignments, resulting in most cases in an enrichment of reference alleles compared to alternative ones. This may occur because reads with true alternative alleles and indels may preferentially be aligned as reference alleles, due to reference bias. This is especially true for some particular cases where the alternative allele is similar to two bases of the reference sequence in the proximity of the DNP (Fig. 1, available as supplementary data at *Bioinformatics Advances* online). The sequencing technology used may enhance this mapping bias. For example, data produced with Ion Torrent technology, which is more prone to indels errors (Bragg *et al.* 2013), may be greatly affected by this issue. To overcome these errors, DNPcall performs the pileup also on the positions directly upstream and downstream the DNP (therefore calling a total of four bases) and removes the reads with indels in them. While this process obviously reduces the number of available reads to perform genotype calling, it greatly enhances its accuracy, especially for heterozygous sites with the characteristic described above (Fig. 1, available as supplementary data at *Bioinformatics Advances* online).

DNPcall is written in R [tested with version 4.2.3, (R Core Team 2023)] and it consists of a single executable R script named DNPcall.R, not requiring installation. Additional software needed are samtools [v1.18 or superior (Li *et al.* 2009, Danecek *et al.* 2021)] and the R packages tidyverse (Wickham *et al.* 2019) and stringr (Wickham *et al.* 2023).

### 2.2 Features and usage

Two input files are necessary for running the pipeline: the list of the BAM files to be analyzed (bamlist) and the list of DNPs to be called. Additionally, the reference genome is needed in FASTA format. The bamlist is a single column file with the path to the BAM files, one per row. The list of DNPs file needs to be structured with the following tab delimited columns: chromosome, position #1 of the DNP, position #2 of the DNP, reference allele, and alternative allele. No header is needed. An example of the formatting of this file can be observed in Table 1, available as supplementary data at *Bioinformatics Advances* online.

DNPcall can be run on the terminal with the command:


Rscript DNPcall.R−bamlist=path_to_list_of_bams−DNPs=path_to_list_of_DNPs−reference=path_to_reference_genome−out=output_name

where path_to_list_of_bams, path_to_list_of_DNPs, path_to_reference_genome and output_name are the aforementioned bamlist, list of DNPs, reference genome and the desired output name, respectively. An optional—*parallel* flag can be included to enable parallel processing. The script will process all the individuals in the bamlist producing individual-specific files with information on the called DNPs (named “full_output_” followed by the name of the sample). These files include DNPs information (chromosome, positions, reference and alternative alleles) followed by the number of reads supporting each genotype, including reads that do not support neither reference nor alternative alleles (N_Other). This information, together with the columns indicating all the genotypes identified (combined_bases) and their respective reads number (combined_counts), can be useful to discriminate between true DNPs and adjacent SNPs and to look for possible issues in the sites or samples analyzed, like triallelic markers and mapping or sequencing errors. This file includes the assigned genotypes (GT field in the form: 0/0 for reference homozygous, 0/1 for heterozygous and 1/1 for alternative homozygous) and the likelihoods of the three possible genotypes (GL0, GL1, GL2) computed with a slightly modified version of the method proposed in Li (2011). In our case, the base quality value was replaced by the mean of the quality of the two bases of the DNP. A genotype is not assigned if too many reads not belonging to either the reference and the alternative alleles are present, in particular if their number is >10% of the reference and alternative total number [therefore if the ratio N_Other/(N_reference+N_alternative) > 0.1]. Finally, two columns (reads_discarded_for_indels and ratio_reads_discarded_for_indels) indicate the number of reads discarded because of indels and their fraction of the total, information that can be useful to identify problematic DNPs and/or regions. An example of this output is shown in Table 2, available as supplementary data at *Bioinformatics Advances* online. DNPcall also produces a summary file named [output_name]_AllGenotypes.txt including the genotypes for each DNP and each individual analyzed. This file includes DNPs information (chromosome, positions, reference and alternative alleles) and the genotypes for each individual (columns named “GT_” followed by the sample names). An example of this file is represented in Table 3, available as supplementary data at *Bioinformatics Advances* online.

Additionally, DNPcall produces plots to better visualize the information included in the tables. For each individual, plots showing the occurrence of the total number of reference, alternative and other reads is produced (named [sample_name]_Ref_read_count.pdf, [sample_name]_Alt_read_count.pdf, and [sample_name]_Other_read_count.pdf, respectively), together with a plot showing cumulatively all the genotypes assigned for that sample ([sample_name]_N_genotypes.pdf) (Fig. 2, available as supplementary data at *Bioinformatics Advances* online). Moreover, an UpSet plot (DNPs_covered_UpSet_plot.pdf) and a bar plot showing the occurrences of unassigned genotypes for DNP (Unassigned_genotypes.pdf) are made to explore problematic DNPs and/or samples (Fig. 3, available as supplementary data at *Bioinformatics Advances* online).

## 3 Results

The We tested DNPcall on the DNPs reported in chromosome 22 in the gnomAD dataset (https://gnomad.broadinstitute.org/data#v2-multi-nucleotide-variants) (Karczewski *et al.* 2020, Wang *et al.* 2020) (Table 1, available as supplementary data at *Bioinformatics Advances* online) on a subset of 13 Western Eurasian individuals from the SGDP dataset (https://reichdata.hms.harvard.edu/pub/datasets/sgdp/) (Mallick *et al.* 2016) (Table 4, available as supplementary data at *Bioinformatics Advances* online). All analyses were performed on a CentOS Linux 7 (Core) server with AMD EPYC 2.9 GHz processors and 32 GB of RAM. The analysis was executed in parallel across the 13 samples, by enabling the—*parallel* option. The entire process required approximately 145 minutes, with peak memory usage of around 3.2 GB per sample.

The list of putative DNPs from the gnomAD was filtered removing repeated two adjacent positions (same positions present in more than one line, keeping only the first one) resulting in a total of 18 782 variants. We identified 10 507 putative DNPs that were successfully called on all the 13 individuals analyzed, indicating particularly reliable positions by the means of coverage and the possibility of being true DNPs. Among them, 802 were found to be polymorphic among the samples analyzed suggesting their possible use for population genetics analysis in an Eurasian context (Table 3, available as supplementary data at *Bioinformatics Advances* online). Conversely, 8275variants returned “NA” in at least one sample. By looking at the individual output files, we explored into more details the reasons for these unassignment. In some of these positions there are samples with a null amount of reads, indicating a region not covered at all possibly due to differences in the quality of the original raw data. More interestingly, there were 7992 positions with at least one sample that shows an allele that is neither the reference nor the alternative one. In most of these cases, there is only one position of the putative DNP that varies with respect to the reference or the alternative allele. Probably these positions do not represent true DNPs, but two adjacent SNPs with reference and alternative alleles differentially distributed among samples and populations, making DNPcall a useful pipeline for discriminating between true DNP and two adjacent SNPs.

DNPcall can be therefore used to accurately call DNP genotypes and, more generally, identify DNPs from a list of two adjacent variant positions. While there are already available tools to finely infer other kinds of microhaplotypes (mostly two SNPs a few base pairs apart), these are specific for microhaplotype of forensic relevance and are not directly applicable to DNPs (Kwon *et al.* 2022, Standage *et al.* 2022). Moreover, the principle behind DNPcall allows it to be potentially applicable, with minimal changes in the source code, to every kind of microhaplotype and, especially, MNVs.

## Supplementary Material

vbaf209_Supplementary_Data

## Data Availability

The source code can be downloaded from https://github.com/fravasini/DNPcall. The gnomAD dataset used to explore DNPs can be downloaded from https://gnomad.broadinstitute.org/data#v2-multi-nucleotide-variants; the SGDP BAM samples used to test DNPcall can be downloaded from https://reichdata.hms.harvard.edu/pub/datasets/sgdp/.
