# Supplementary material for: DNPcall: a new pipeline for accurate double nucleotide polymorphism calling
Source: Bioinform Adv. 2025 Sep 5;5(1):vbaf209. doi: 10.1093/bioadv/vbaf209 (PMC12502915; doi:10.1093/bioadv/vbaf209)
Supplement: vbaf209_Supplementary_Data [file vbaf209_supplementary_data.zip › Supplementary_figure.pdf]

Supplementary figures

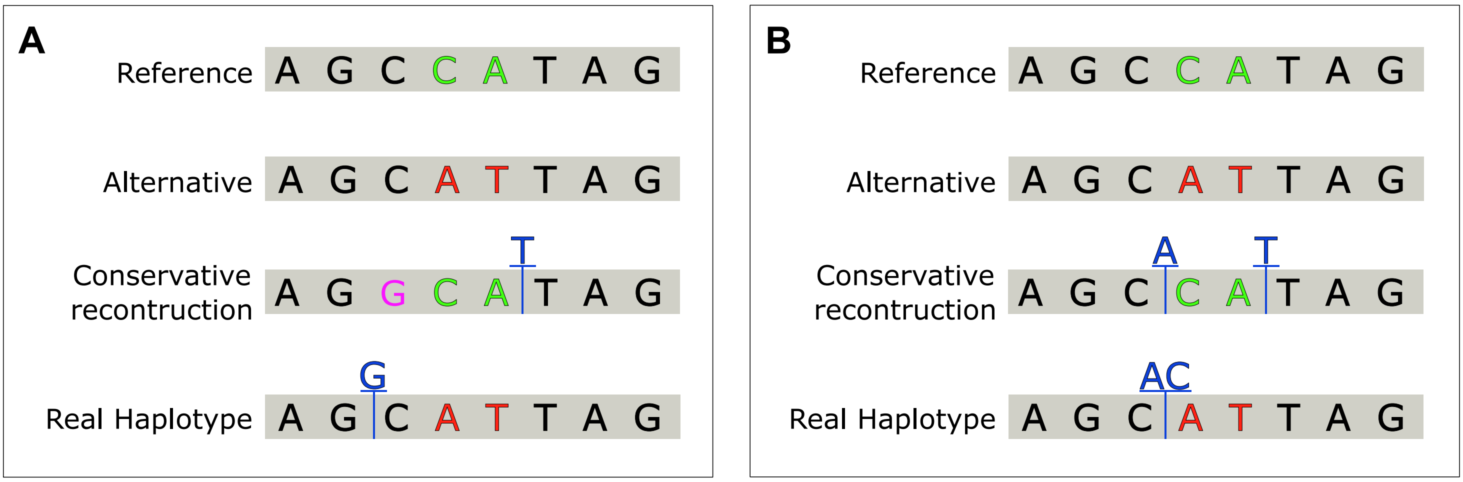

Supplementary figure S1. Examples of possible problems in the mapping of reads carrying alternative alleles due to the nature of the sequence nearby the DNP. When an indel (real or caused by sequencing error) is present in the read, the mapping process may preferiantially assign a reference allele because it allows for less mismatches. A) Situation with a single inserted base resulting in a false reference allele in the DNP and a false variant in the position upstream. B) Situation with two inserted bases resulting in a false reference allele and wrong positioning of the putative insertions.

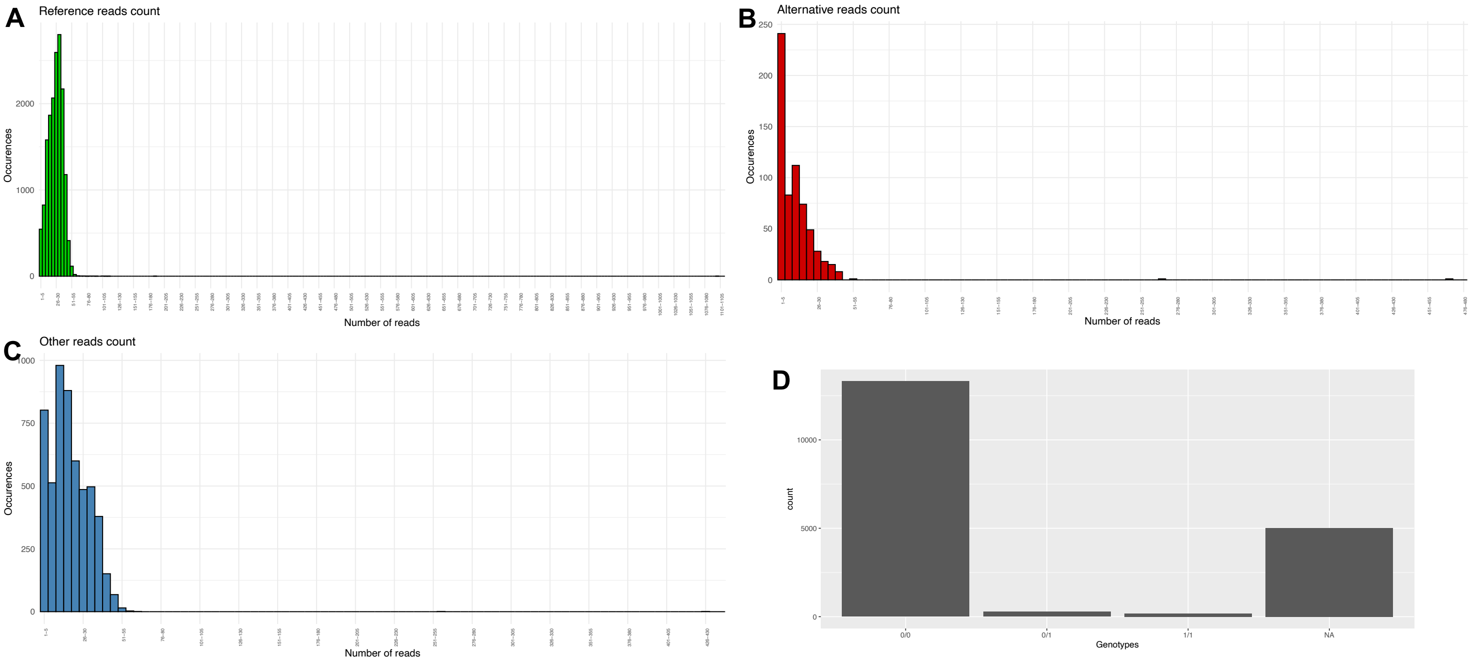

Supplementary figure S2. Examples of individual output plots produced by DNPcall. Plots A, B and C represent the total occurrences of reference, alternative and other (i.e., neither reference nor alternative) reads, respectively. The long right tails indicate that at least one DNP is covered by a high number of reads, probably inidcating a region with mapping issues. D) Total occurences of the possible genotypes in the sample (0/0, homozygous reference; 0/1 heterozygous; 1/1, homozygous alternative.

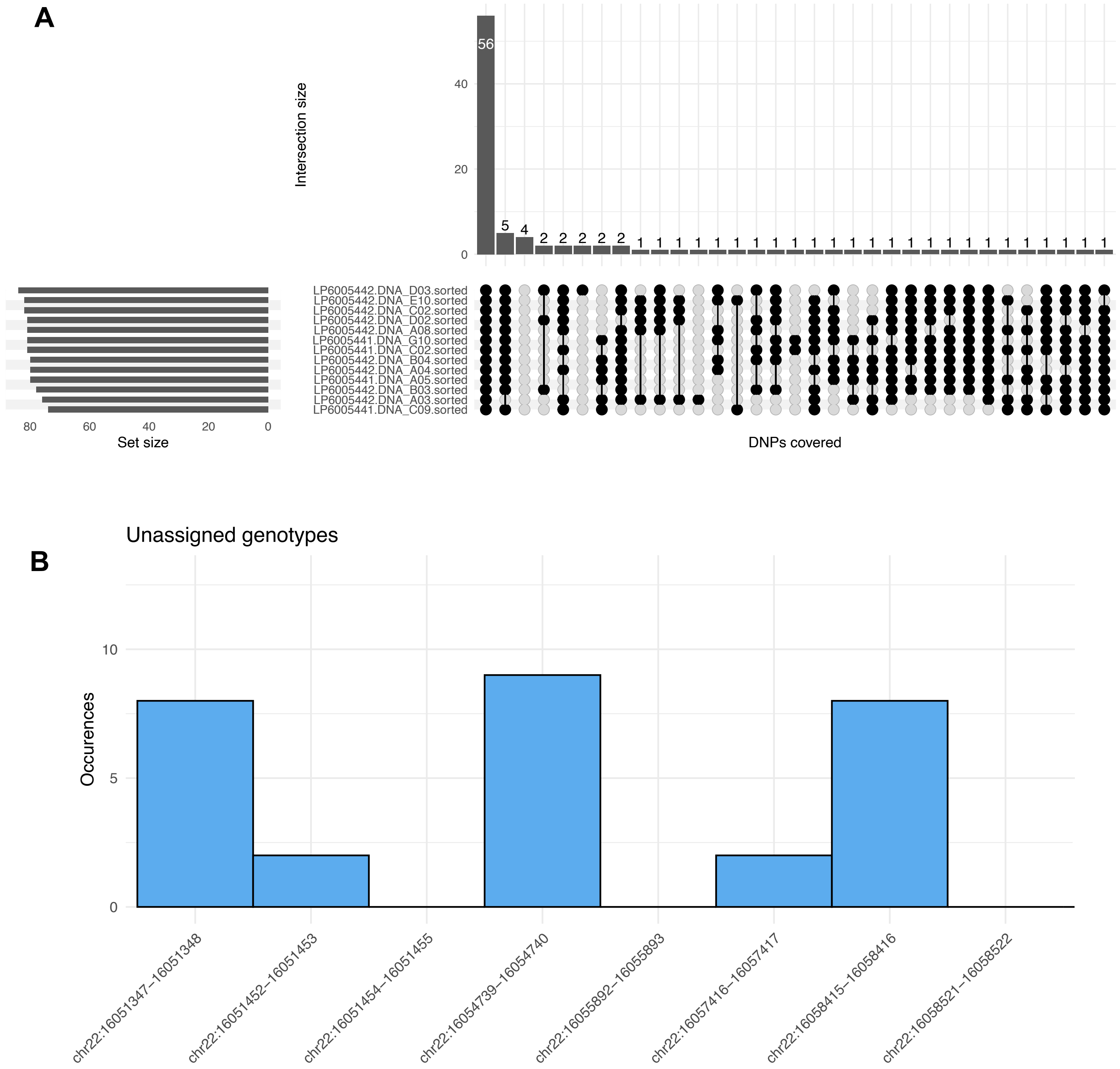

Supplementary figure S3. Examples of summary plots. A) UpSet plot showing DNP coverage among samples. B) Bar plot indicating the occurences of unassigned genotypes (NA) for each DNP. These plots are made with a subset of DNPs because the originals with the SGDP data used, due to their large size, cannot be represented properly here. They can be found at https://github.com/fravasini/DNPcall.
